# A nationwide population-based study of incidence and mortality of lung cancer in idiopathic pulmonary fibrosis

**DOI:** 10.1038/s41598-021-82182-8

**Published:** 2021-01-28

**Authors:** Myung Jin Song, Song Yee Kim, Moo Suk Park, Min Jin Kang, Sang Hoon Lee, Seon Cheol Park

**Affiliations:** 1 Division of Pulmonary and Critical Care Medicine, Department of Internal Medicine, Seoul National University College of Medicine, Seoul National University Bundang Hospital, Seongnam, South Korea; 2 Division of Pulmonology, Department of Internal Medicine, Institute of Chest Diseases, Severance Hospital, Yonsei University College of Medicine, 50-1 Yonsei-ro, Seodaemun-gu, Seoul, 03722 Republic of Korea; 3 Research Institute, National Health Insurance Service Ilsan Hospital, Goyang-si, Gyeonggi-do Republic of Korea; 4 Division of Pulmonology, Department of Internal Medicine, National Health Insurance Service Ilsan Hospital, Ilsan-ro 100, Ilsandong-gu, Goyang-si, Gyeonggi-do Republic of Korea

**Keywords:** Oncology, Risk factors

## Abstract

Idiopathic pulmonary fibrosis (IPF) is an independent risk factor for lung cancer (LC) development; however, there are currently no clinical guidelines for LC surveillance in IPF. This study aimed to investigate the cumulative incidence and survival outcomes of LC in IPF. Using the National Health Insurance Service database, including medical information on people aged ≥ 40 years between 2011 and 2016, we identified IPF patients and confirmed the presence of comorbid LC. Patients diagnosed with IPF in 2011 were washed out, and mortality data were analyzed from 2012 to 2018. A total of 7277 newly diagnosed IPF patients were identified among Korean citizens aged ≥ 40 years (about 50 million people) between 2011 and 2016. Their average age was 71.5 years and 72.8% of them were male. The prevalence of LC in the IPF cases was 6.4%. The cumulative incidence rates of LC in IPF patients who did not have LC at the time of IPF diagnosis were 1.7%, 4.7%, and 7.0%, at 1, 3, and 5 years, respectively. The median time from IPF diagnosis to LC development was 16.3 (Interquartile range, 8.2–28.8) months. The survival rate was significantly lower in the IPF with LC group than the IPF without LC group (*P* < 0.001). We concluded that IPF increases LC risk, and LC weakens survival outcomes in IPF. Close surveillance for LC development is mandatory for patients with IPF.

## Introduction

Idiopathic pulmonary fibrosis (IPF) is the most commonly occurring type of idiopathic interstitial pneumonia, characterized by chronic, progressive, fibrotic interstitial lung disease of an unknown cause and a histopathological pattern of usual interstitial pneumonia^1,2^. Epidemiological studies have revealed that IPF is an independent risk factor for lung cancer (LC) development after adjusting for shared risk factors. Hubbard et al. reported that the relative risk of developing LC in IPF is approximately eight times higher than in the general population, after adjusting for smoking history, based on United Kingdom healthcare databases^3^. The prevalence of LC in IPF was reported to be 4.4–13%, and up to 48.2% in an autopsy study of 83 usual interstitial pneumonia patients^3–9^. LC in IPF occurs more frequently in older male smokers and patients with combined emphysema^7,8,10^. Comorbid LC significantly affects survival in IPF. The mortality values are higher among IPF patients with LC than IPF patients without it^11–13^ and those with LC in the general population^8,14^. Additionally, the treatment of LC itself can cause acute IPF exacerbation, which is the most lethal life-threatening complication of IPF^15–17^.

Despite the presence of notable epidemiologic associations between LC and IPF, and poor survival outcomes, there are currently no clinical guidelines for the screening of LC in IPF; since the natural history of IPF is highly variable, some patients show a relatively stable disease course without symptoms and, therefore, do not routinely visit hospitals^18^. In the absence of an official recommendation for LC screening in IPF, a large number of IPF patients are diagnosed with advanced LC at the time of hospital visit due to symptom development over several years.

In this study, we investigated the demographic characteristics, cumulative incidence, and mortality associated with LC in IPF patients using nationwide data to suggest the direction of LC surveillance.

## Results

### Patient characteristics

A total of 7277 newly diagnosed IPF patients were identified between 2012 and 2016. Their average age was 71.5 years and 72.8% of the study population was male. Of the IPF patients, 464 (6.4%) were diagnosed with LC during the study period. The baseline demographics of the IPF patients with and without LC (IPF with LC and IPF without LC groups) were compared (Table 1). The proportion of men was lower (74.4% vs. 92.6%; *P* < 0.001) and average age was higher (71.5 vs. 70.6 years; *P* = 0.029) in the IPF without LC group than in the IPF with LC group. Similar proportions of urban and rural area-dwelling patients were observed in both the IPF without LC and IPF with LC groups. More than 40% of the patients in both groups were included in the fifth quintile of household income, the lowest socioeconomic status, and the distributions of household income were similar between the groups. Of the 464 patients in the IPF with LC group, 254 (54.7%) were diagnosed with IPF before LC, 127 (27.4%) had a simultaneous diagnosis of IPF and LC, and 83 (17.9%) were diagnosed with LC before IPF. Patients were classified as having a “simultaneous diagnosis” if LC was diagnosed within 3 months of IPF being diagnosed. Patients diagnosed with IPF more than three months in advance of the LC diagnosis were classified into the “IPF in advance” group, while those who were diagnosed with LC more than three months in advance of IPF were classified into the “LC in advance” group. The baseline demographics of the IPF with LC group, according to the order of diagnosis, are shown in Supplementary Table S1. There was no significant difference in the baseline demographics across the three groups, including age, sex, region of residence, and household income.

**Table 1:** Baseline characteristics of IPF patients with or without LC, 2012–2016.

	Total	IPF without LC	IPF with LC	*P* value
Total, n (%)	7277 (100.0%)	6813 (93.6%)	464 (6.4%)	
Male, n (%)	5499 (72.8%)	5069 (74.4%)	430 (92.6%)	< 0.001
**Age**				
Mean	71.5 ± 9.0	71.5 ± 9.0	70.6 ± 8.0	0.029
40–49 years	91 (1.3%)	88 (1.3%)	3 (0.6%)	< 0.001
50–59 years	628 (8.6%)	593 (8.7%)	35 (7.5%)	
60–69 years	2098 (28.8%)	1933 (28.4%)	165 (35.6%)	
70–79 years	3132 (43.0%)	2924 (42.9%)	208 (44.8%)	
≥ 80 years	1328 (18.3%)	1275 (18.7%)	53 (11.4%)	
**Region of residence, n (%)**				
Urban	3262 (44.8%)	3040 (44.6%)	222 (47.8%)	0.177
Rural	4015 (55.2%)	3773 (55.4%)	242 (52.2%)	
**Household income**^**a**^**, n (%)**				
1st quintile	1193 (16.4%)	1137 (16.7%)	56 (12.1%)	0.089
2nd quintile	850 (11.7%)	795 (11.7%)	55 (11.8%)	
3rd quintile	903 (12.4%)	849 (12.5%)	54 (11.6%)	
4th quintile	1315 (18.1%)	1227 (18.0%)	88 (19.0%)	
5th quintile	3016 (41.4%)	2805 (41.2%)	211 (45.5%)	

^a^Household income decreases from the 1st to the 5th quintile.

Values are expressed as means ± standard deviations or numbers (%).

*IPF* idiopathic pulmonary fibrosis, *LC* lung cancer.

### Incidence of IPF and IPF with LC

**Figure 1:**
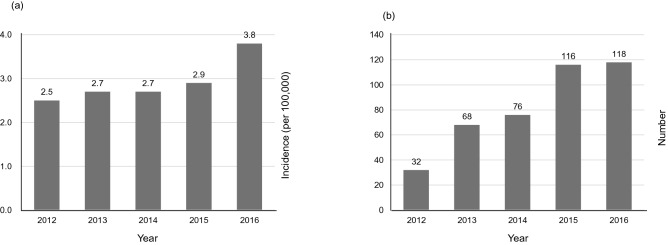
Incidence of IPF and the number of IPF with LC patients between 2012 and 2016. (**a**) Incidence of IPF. (**b**) The number of IPF with LC patients. *IPF* idiopathic pulmonary fibrosis, *LC* lung cancer.

**Figure 2:**
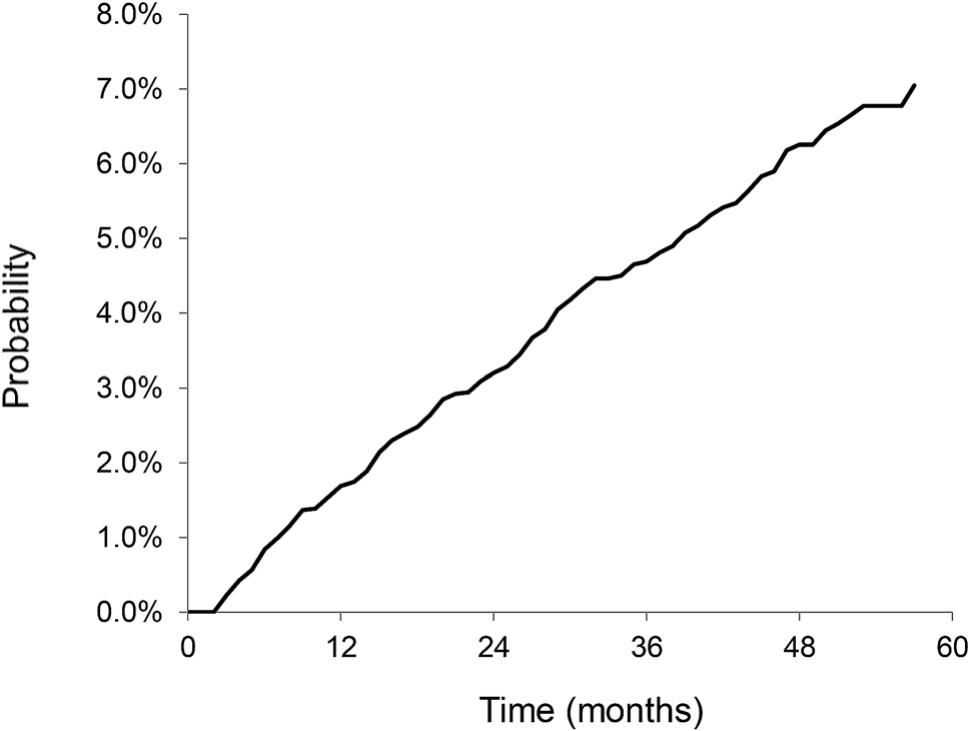
Cumulative incidence of LC in IPF patients. *IPF* idiopathic pulmonary fibrosis, *LC* lung cancer.

Between 2012 and 2016, the incidence of IPF increased. In 2012, the incidence of IPF was 2.5 per 100,000 population in Koreans aged ≥ 40 years; this value increased to 3.8 per 100,000 in 2016. The number of IPF with LC patients was 32 cases in 2012 and 118 cases in 2016 (Fig. 1). The cumulative incidence rates of LC in the IPF patients who did not have LC at the time of IPF diagnosis were 1.7%, 4.7%, and 7.0% at 1, 3, and 5 years, respectively (Fig. 2). The median time from IPF diagnosis to LC development was 16.3 (Interquartile range [IQR], 8.2–28.8) months. In the comparison conducted by age group, the median time from IPF diagnosis to LC development was not significantly different (*P* = 0.852, Table 2).

**Table 2:** Time from IPF diagnosis to LC development according to age group.

	N	Time (months)	*P* value
Total	254	16.3 (8.2–28.8)	0.852
40–59 years	21	16.4 (63.8–29.8)	
60–69 years	95	18.9 (9.3–28.0)	
70–79 years	116	15.7 (7.9–28.9)	
≥ 80 years	22	14.3 (7.0–27.9)	

Values are expressed as medians (interquartile range).

*IPF* idiopathic pulmonary fibrosis, *LC* lung cancer.

### Mortality

**Figure 3:**
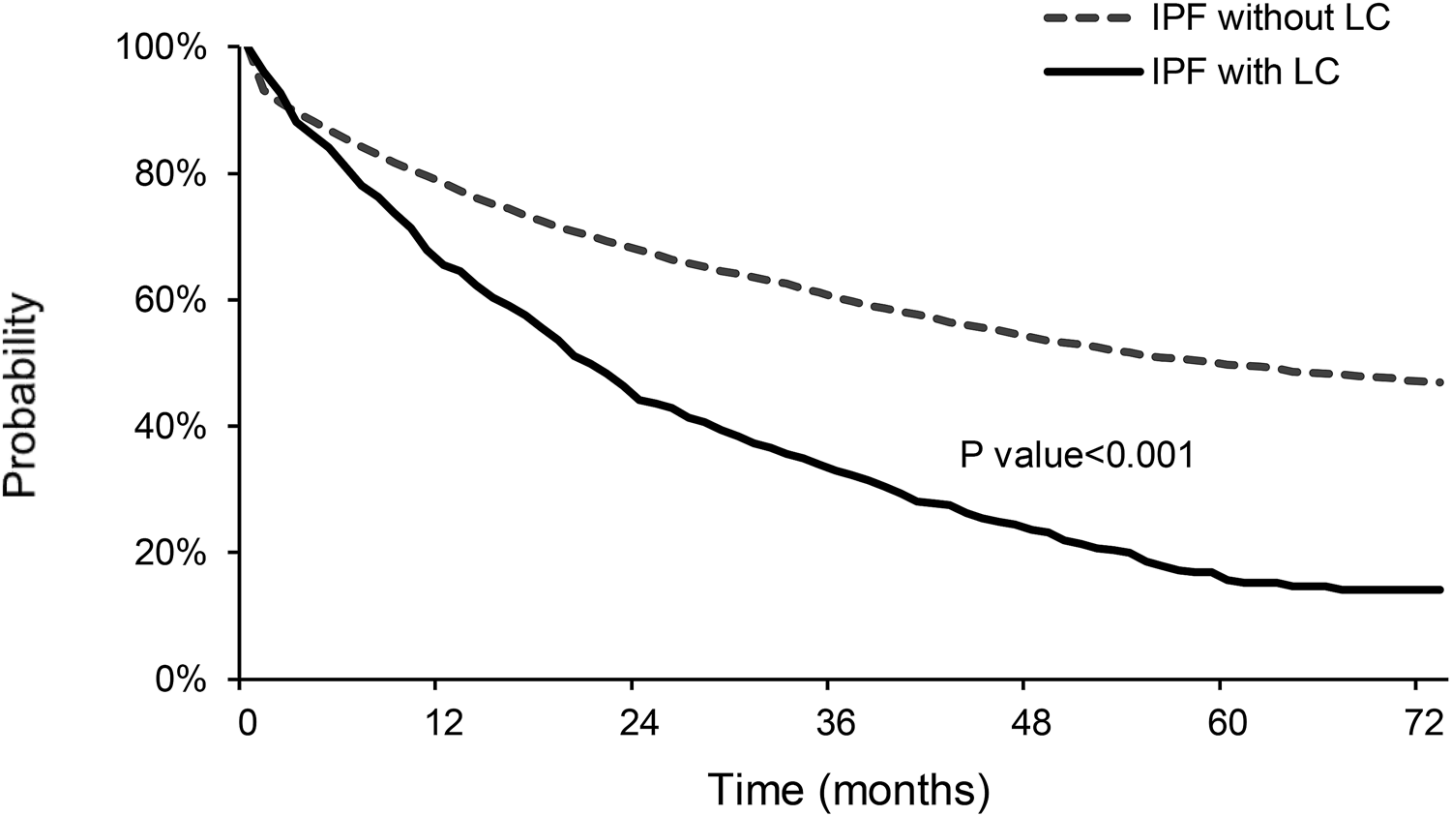
Kaplan–Meier survival curves of all-cause mortality in IPF with LC, and IPF without LC. *IPF* idiopathic pulmonary fibrosis, *LC* lung cancer.

In the total 7277 IPF patients, 1- and 2-year survival rates were 77.7% and 66.3%, respectively. In Kaplan–Meier analysis, survival rates were significantly lower in the IPF with LC group than in the IPF without LC group [log-rank test, *P* < 0.001; Fig. 3]: 1- and 2-year survival rates were 65.5% and 44.2% in the IPF with LC group and 78.5% and 67.8% in the IPF without LC group, respectively. In multivariate Cox proportional hazard analysis, the presence of lung cancer (hazard ratio [HR] 2.922, *P* < 0.001) and age at the diagnosis of IPF (HR 1.042, *P* < 0.001) were independent risk factors for mortality in IPF patients (Table 3).

**Table 3:** Cox proportional hazard regression analysis for mortality in IPF patients.

	HR (95% CI)	*P* value
Lung cancer	2.922 (2.054–2.557)	< 0.001
Age (year)	1.042 (1.038–1.046)	< 0.001
Sex (male)	0.932 (0.862–1.008)	0.079
Region of residence (urban)	0.936 (0.874–1.001)	0.054

*CI* confidential interval, *HR* hazard ratio.

## Discussion

In the present study, we estimated the cumulative incidence of LC in IPF patients and mortality associated LC in IPF patients using a nationwide database. Our data showed that the cumulative incidence rates of LC in IPF patients who did not have LC at IPF diagnosis were 1.7%, 4.7%, and 7.0% at 1, 3, and 5 years, respectively. The median time from IPF diagnosis to LC development was 16.3 months. The 1- and 2-year survival rates were 65.5% and 44.2% in the IPF with LC group, which were significantly lower than those in the IPF without LC group. This study is meaningful as the largest to estimate the cumulative incidence of LC in IPF using nationwide data.

Although epidemiological studies have revealed that IPF is an independent risk factor for LC development, the pathogenesis connecting the two diseases is poorly understood. According to the existing literature, LC and IPF share the following pathogenetic mechanisms in terms of their development: genetic alterations including micro-satellite instability, loss of gene heterozygosity and gene mutations including those of p53; alteration of epigenetics; abnormal expression of microRNAs; cellular and molecular aberrances such as an altered response to regulatory signals and reduced cell-to-cell communication; and activation of specific signaling transduction pathways^19–22^. The most frequently observed histologic LC type in IPF patients was squamous cell carcinoma followed by adenocarcinoma; adenocarcinoma is the most commonly observed histologic type in the general population^6–8,10–13,23^. LC in IPF is typically observed in the peripheral zone of the lower lung in the fibrotic area^24^.

Few studies have reported on the incidence of LC during the follow-up period for IPF. Ozawa et al. reported cumulative LC incidence rates of 3.3%, 15.4%, and 54.7% at 1, 5, and 10 years, respectively, in 103 IPF patients^10^. Kato et al. demonstrated incidences of 12.2% and 23.3% at 5 and 10 years, respectively, in 632 IPF patients^7^. Tomasetti et al. showed that among 181 IPF patients, 16 were diagnosed with LC during the follow-up period for IPF. In that study, the median duration from IPF diagnosis to LC development was 30.0 (IQR, 27.5–84.1) months, and 81.8% of the IPF patients with LC developed LC in 3 years from IPF diagnosis^8^. However, each of the three aforementioned studies was conducted at a single center. Yoon et al. conducted a study based on interstitial lung disease registry data in the United States, which included 1108 IPF patients, and reported that the median time from IPF diagnosis to LC development was 53.0 (IQR, 25.0–77.0) months, and the incidence of LC increased in the first two years after IPF diagnosis, which persisted until year four^13^. The present study is the largest nationwide cohort study to date based on the NIHS database of almost all South Korean citizens aged ≥ 40 years (about 50 million people) and included 7277 IPF patients from this population. In this study, the cumulative incidence rates of LC in IPF at 1, 3, and 5 years of 1.7%, 4.7%, and 7.0%, respectively, were lower than those observed in previous studies. IPF shows a varied natural course after diagnosis^18^; therefore, patients who visit hospitals routinely and are enrolled in study registries from previous studies may have a relatively severe and rapid progressive disease course. This may be the reason for the lower incidence observed in our population-based study, which is based on the NHIS database of almost all Korean citizens, and also a strength of this study compared to previous studies.

The cumulative incidence graph showed a constant incidence rate over the 5-year study period, while previous studies reported that the incidence rate increased until 3–4 years from IPF diagnosis and then decreased^8,13^. The median time from IPF diagnosis to LC development tended to be shorter in those aged ≥ 60 years, but there was no significant difference between the age groups.

Although there is no official guideline for LC surveillance till date, various suggestions have been proposed in previous reviews. Tzouvelekis suggested the performance of high-resolution computed tomography (HRCT) once a year in all IPF patients. For nodules with a diameter smaller than 8 mm, HRCT is suggested every 3–6 months. If HRCT shows progression of the nodule, and for nodules with a diameter of at least 8 mm, positron emission tomography-CT is recommended^25^. Lederer et al., in another review, proposed that annual low-dose CT should be performed according to the U.S. Preventive Services Task Force criteria for LC screening, and nodules should be managed according to risk group based on the established guidelines of the Fleischner Society^1^.

Considering previous reports, which state that the LC incidence in IPF patients is approximately eight times higher than in the general population after adjusting for smoking history^3^, and the fact that the cumulative incidence rate was kept constant during the study period, it is suggested that all IPF patients undergo close HRCT screening.

Another challenge associated with IPF in LC, in real clinical practice, is the identification of whether the antifibrotic agents that are used to reduce the degree of forced vital capacity decline in IPF patients have anti-cancer effects. Nintedanib and pirfenidone are two antifibrotic agents that have been approved for IPF treatment. A preclinical experimental study revealed that a combination of cisplatin and pirfenidone increases cell death rates and decreases cancer progression rates^26^. A retrospective study reported that the incidence of LC was lower in IPF patients receiving pirfenidone^27^. Nintedanib was first approved in combination with docetaxel as a second line treatment for advanced non-small cell LC^28^. The anti-cancer effect of these two antifibrotic agents should be further investigated in both therapeutic and protective aspects.

This study has several limitations. First, we defined IPF using the ICD-10 code assigned by healthcare providers; this may have reduced the diagnostic accuracy owing to inconsistencies with the diagnostic criteria defined by the International Consensus Statement of the American Thoracic Society and European Respiratory Society in 2011^29^. However, IPF is classified as a rare intractable disease in Korea and strictly controlled by the Korean NHIS for medical cost reductions. This may lead physicians to enter the diagnostic code with care. Second, data on confounding factors that may have affected the incidence and mortality of both IPF and LC, such as smoking, pulmonary function (forced expiratory volume in one second and forced vital capacity), and concomitant emphysema, were not available in the study. Further study is needed to confirm whether close HRCT surveillance for LC in IPF will help improve the mortality of IPF patients with LC.

In conclusion, the present study revealed that the prevalence of LC in IPF patients was 6.4% and the cumulative incidence rates were 1.7%, 4.7%, and 7.0% at 1, 3, and 5 years, respectively, after IPF diagnosis. The 5-year survival rate was 15.6% in the IPF with LC group, which was significantly lower than that in the IPF without LC group. Our findings suggest the need for close HRCT surveillance for LC in all IPF patients.

## Methods

### Data source

The National Health Insurance Service (NHIS) in Korea has provided health insurance services to all Korean citizens living in Korea since 2000. Consequently, a large amount of health-related data has accumulated in the NHIS database. To offer relevant and useful data for health researchers, the NHIS established a population database including data on socioeconomic status (household income), medical treatments, medical care institutions, and general health examinations since 2002. From the NHIS database, we identified IPF and LC based on International Classification of Disease, Tenth Revision (ICD-10) diagnostic codes. This study was approved by the Institutional Review Board and Ethics Committee of National Health Insurance Service of the Ilsan Hospital (IRB number: NHIMC 2019-1-291). All methods were performed in accordance with the Declaration of Helsinki.

### Case identification

The NHIS database includes medical data on almost all South Korean citizens aged ≥ 40 years (about 50 million people) between 2011 and 2016. From this population, we identified patients visiting medical institutions with a diagnosis of IPF, coded as J84.18 according to the ICD-10. We excluded patients with ICD-10 codes for both IPF and connective tissue disease, owing to the possibility of interstitial lung disease being related to connective tissue disease. The ICD-10 codes for connective tissue disease were as follows: M05 for rheumatoid arthritis, M07 for psoriatic and enteropathic arthropathies, M30 for polyarteritis, M31 for other necrotizing vasculopathies, M32 for systemic lupus erythematosus, M33 for dermatopolymyositis, M34 for systemic sclerosis, M35 for other systemic involvement of connective tissue, and M45 for ankylosing spondylitis. The code J84.18 was classified as a rare intractable disease, and patients assigned to this code receive medical cost reductions from the NHIS by up to 10% of the total cost. Owing to the financial problems associated with rare intractable diseases, the ICD for these diseases has high sensitivity and specificity^30^. The ICD-10 code C34 was used for malignant neoplasms of the bronchus and lung. To estimate the cumulative incidence of LC in patients with newly diagnosed IPF and the time from IPF diagnosis to LC development, we excluded patients who were diagnosed with IPF in 2011 and analyzed those who were newly diagnosed with IPF after 2012. Mortality data were analyzed from 2012 to 2018.

### Statistical analysis

Continuous variables were analyzed using the Student’s t-test, and categorical variables using Chi-squared distribution. Cumulative time-to-event distributions were estimated using the Kaplan–Meier method. Log-rank test was used to compare survival curves, and Cox proportional hazards regression analyses were conducted to assess the impact of LC on survival in IPF. In all cases, *P* values < 0.05 were considered statistically significant. All statistical analyses were performed using the SAS program, version 9.4 (SAS Institute, Cary, NC, USA).

### Ethics approval and consent to participate

This study was approved by the Institutional Review Board and Ethics Committee of National Health Insurance Service of the Ilsan Hospital (IRB number: NHIMC 2019-1-291). Written informed consent was waived as the nature of retrospective study by IRB.

## Supplementary Information

Supplementary Table S1.

## Data Availability

The datasets used and analyzed in the current study are available from the corresponding author on reasonable request.

## Abbreviations

HRCT: High-resolution computed tomography
ICD-10: International Classification of Disease, Tenth Revision
IPF: Idiopathic pulmonary fibrosis
LC: Lung cancer
NHIS: National Health Insurance Service
