# Supplementary material for: A nationwide population-based study of incidence and mortality of lung cancer in idiopathic pulmonary fibrosis
Source: Sci Rep. 2021 Jan 28;11:2596. doi: 10.1038/s41598-021-82182-8 (PMC7843601; doi:10.1038/s41598-021-82182-8)
Supplement: Supplementary file 1 — Supplementary Table S1. [file 41598_2021_82182_MOESM1_ESM.docx]

**A nationwide population-based study of incidence and mortality of lung cancer in idiopathic pulmonary fibrosis**

Myung Jin Song^1^, Song Yee Kim^2^, Moo Suk Park^2^, Min Jin Kang^3^, Sang Hoon Lee^2,*^, Seon Cheol Park^4,*^

^1^Division of Pulmonary and Critical Care Medicine, Department of Internal Medicine, Seoul National University College of Medicine, Seoul National University Bundang Hospital, Seongnam, South Korea

^2^Division of Pulmonology, Department of Internal Medicine, Institute of Chest Diseases, Severance Hospital, Yonsei University College of Medicine, Seoul, Republic of Korea

^3^Research Institute, National Health Insurance Service Ilsan Hospital, Goyang-si, Gyeonggi-do, Republic of Korea

^4^Division of Pulmonology, Department of Internal Medicine, National Health Insurance Service Ilsan Hospital, Goyang, Gyeonggi-do, Republic of Korea

^*^These authors contributed equally to this work.

**Corresponding author:**

Seon Cheol Park, MD, PhD

Division of Pulmonology, Department of Internal Medicine, National Health Insurance Service Ilsan Hospital, Ilsan-ro 100, Ilsandong-gu, Goyang-si, Gyeonggi-do, Republic of Korea

E-mail address: [parksc@nhimc.or.kr](mailto:parksc@nhimc.or.kr)

**Co-author corresponding author**

Sang Hoon Lee, MD, PhD

Division of Pulmonology, Department of Internal Medicine, Institute of Chest Diseases, Severance Hospital, Yonsei University College of Medicine, 50-1 Yonsei-ro, Seodaemun-gu, Seoul 03722, Republic of Korea

E-mail address: cloud9@yuhs.ac

Supplementary Table S1. Baseline characteristics of IPF patients with LC, according to the order of diagnosis

|  |  | IPF in advance | Simultaneous diagnosis | LC in advance | P value |
| --- | --- | --- | --- | --- | --- |
| Total, n (%) | | 254 (54.7%) | 127 (27.4%) | 83 (17.9%) |  |
| Male, n (%) | | 236 (92.1%) | 117 (92.1%) | 77 (92.8%) | 0.961 |
| Age | |  |  |  |  |
|  | Mean | 70.0 ± 7.8 | 71.3 ± 7.8 | 71.3 ± 8.7 | 0.223 |
|  | 40–49 years | 1 (0.4%) | 1 (0.8%) | 1 (1.2%) | 0.648 |
|  | 50–59 years | 20 (7.9%) | 8 (6.3%) | 7 (8.4%) |  |
|  | 60–69 years | 95 (37.4%) | 44 (34.6%) | 26 (31.3%) |  |
|  | 70–79 years | 116 (45.7%) | 54 (42.5%) | 38 (45.8%) |  |
|  | ≥80 years | 22 (8.6%) | 20 (15.8%) | 11 (13.3%) |  |
| Region of residence, n (%) | | |  |  |  |
|  | Urban | 117 (46.1%) | 65 (51.2%) | 40 (48.2%) | 0.640 |
|  | Rural | 137 (53.9%) | 62 (28.8%) | 43 (51.8%) |  |
| Household income^a^, n (%) | | |  |  |  |
|  | 1^st^ quintile | 33 (13.0%) | 15 (11.8%) | 8 (9.6%) | 0.149 |
|  | 2^nd^ quintile | 31 (12.2%) | 18 (14.2%) | 6 (7.3%) |  |
|  | 3^rd^ quintile | 31 (12.2%) | 14 (11.0%) | 9 (10.8%) |  |
|  | 4^th^ quintile | 53 (20.9%) | 26 (20.5%) | 9 (10.8%) |  |
|  | 5^th^ quintile | 106 (41.7%) | 54 (42.5%) | 51 (61.5%) |  |

^a^Household income decreases from the 1^st^ to the 5^th^ quintile.

Values are expressed as mean ± standard deviation or number (%)

IPF, idiopathic pulmonary fibrosis; LC, lung cancer
